# A Novel Zernike Moment-Based Real-Time Head Pose and Gaze Estimation Framework for Accuracy-Sensitive Applications

**DOI:** 10.3390/s22218449

**Published:** 2022-11-03

**Authors:** Hima Deepthi Vankayalapati, Swarna Kuchibhotla, Mohan Sai Kumar Chadalavada, Shashi Kant Dargar, Koteswara Rao Anne, Kyamakya Kyandoghere

**Affiliations:** 1 Department of Electronics and Communication Engineering, Kalasalingam Academy of Research and Education, Krishnankovil 626126, India; 2 Department of Computer Science and Engineering, Koneru Lakshmaiah Education Foundation, Vaddeswaram 522302, India; 3 Department of Electronics and Communication Engineering, VelTech Rangarajan Dr. Sagunthala R&D Institute of Science and Technology, Chennai 600062, India; 4 Department of Computer Science and Engineering, Kalasalingam Academy of Research and Education, Krishnankovil 626126, India; 5 Institute for Smart Systems Technologies, University Klagenfurt, 9020 Klagenfurt am Wörthersee, Austria

**Keywords:** head pose and gaze estimation (HPGE), feature extraction, Zernike moments, principal component analysis, linear discriminant analysis

## Abstract

A real-time head pose and gaze estimation (HPGE) algorithm has excellent potential for technological advancements either in human–machine or human–robot interactions. For example, in high-accuracy advent applications such as Driver’s Assistance System (DAS), HPGE plays a crucial role in omitting accidents and road hazards. In this paper, the authors propose a new hybrid framework for improved estimation by combining both the appearance and geometric-based conventional methods to extract local and global features. Therefore, the Zernike moments algorithm has been prominent in extracting rotation, scale, and illumination invariant features. Later, conventional discriminant algorithms were used to classify the head poses and gaze direction. Furthermore, the experiments were performed on standard datasets and real-time images to analyze the accuracy of the proposed algorithm. As a result, the proposed framework has immediately estimated the range of direction changes under different illumination conditions. We obtained an accuracy of ~85%; the average response time was 21.52 and 7.483 ms for estimating head poses and gaze, respectively, independent of illumination, background, and occlusion. The proposed method is promising for future developments of a robust system that is invariant even to blurring conditions and thus reaching much more significant performance enhancement.

## 1. Introduction

The head pose and gaze estimation (HPGE) algorithm plays a vital role in identifying a person’s direction of observation and attention while driving. Safe driving is a complex task that needs a wide range of abilities and skills in cognitive, physics, and sensory data [1]. Driving does not always occur in ideal conditions, such as being well rested, well trained, and not engaged with non-driving-related activities. According to road accident statistics, the estimation of driver distraction can help improve the country’s economic status [2], and HPGE plays a very crucial step in developing Advanced Driver Assistance System (ADAS).

Distraction is considered as the diversion state of the person concentrating on a situation and object unrelated to the primary task, which leads to risky decisions in the real-time environment. The head pose estimation calculates the spatial location of the human head along with the position or rotation angle (front or side poses) in an image. Gaze estimation measures eyeball direction to predict the human attention level and better understand human activities. Head pose always coincides with gaze directions to know the focus of attention. It also successfully provides robust and accurate perception to interact with humans [1]. It is mainly used for analyzing complex meaningful gestures such as head nodding or pointing gestures. Gaze direction is achieved by capturing and analyzing the appearance of human eye images. The eye is one of the major sensory organs that conveys reliable information to the brain to perform tasks.

The main objective of this work is to develop a framework for monitoring the attentiveness and behavior of the person by estimating the head pose and gaze directions. This information is used to analyze and understand the attitude (behavior) of the person [3]. Therefore, we need to adopt a technique that is not intrusive and should not cause inconvenience to the person for developing an inexpensive automatic Head Pose and Gaze Estimation system, which is essential to understand the attention and behavior of the person. The automatic behavior identification system is crucial in real-time applications such as psychology, biometrics, surveillance, and safety surveillance systems. The HPGE system’s performance is analyzed using the elapsed time and recognition accuracy.

This paper involves and analyzes the various head pose and gaze estimation techniques that exist in the literature. We have analyzed the parameters and constraints in the existing HPGE systems and propose a hybrid Zernike moment-based framework to overcome the drawbacks. It also includes a detailed methodology with the experimental results on standard databases and images acquired in real time (which consist of facial images with different sizes and illumination conditions).

## 2. Related Work

This section analyzes the state of the art in estimating attentiveness by focusing on conventional appearance-based methods to recent advanced deep learning algorithms for: (1) head pose estimation (2) gaze estimation, and (3) an integrated HPGE system on images in constrained and unconstrained environments.

### 2.1. Appearance and Template-Based Methods

In appearance-based methods, each train and test image is projected into the high dimensional feature space, and the distance between the features is calculated to find the best match. These methods extract the local features from the input image, invariant to varying illuminations [4]. An extension to these methods is template matching, which can be adapted and trained based on our application at varying environmental conditions. The performance of these methods depends on the size of the training data, occlusions, and the redundancy of input data [5,6]. Most of these methods calculate the best match using a minimum distance metric to find head pose and gaze orientations or movements. The minimum distance metric classifier is based on either Euclidean distance or Mahalanobis distances. Euclidean distance is the distance between two points x and y, represented as Equation (1)

$$\mathrm{dist}\left(x,y\right)=\mathrm{sqrt}\left(\left(x-y\right){\left(x-y\right)}^{T}\right)$$

(or)

(1) $$\mathrm{dist}\left(x,y\right)=\mathrm{sqrt}(\overset{2}{\underset{i=1}{\sum }}{\left(\left|x_{i}\left|-\right|y_{i}\right|\right)}^{2})$$

Mahalanobis distance is calculated as Equation (2)

(2) $${\left|\left|x-y\right|\right|}_{\left(\sum i\right)}^{2}={\left(x-y\right)}^{T}{\left(\sum i\right)}^{-1}\left(x-y\right)$$

To overcome the training data size and redundancy drawback, dimensionality reduction techniques are performed on the extracted feature vector. Dimensionality reduction techniques are mainly classified into (i) principal component analysis (PCA), (ii) Kernel PCA, (iii) local discriminant analysis (LDA), (iv) canonical correlation Analysis (CCA), (iv) independent component analysis (ICA), (v) Isomaps, (vi) diffusion maps, etc.

According to the literature, appearance-based methods use an entire eye image as a high dimensional input feature vector and map them to a low dimensional gaze directional space vector. Therefore, these methods do not require extracting the eye features and giving accurate results, and the performance depends on the number of training samples [4,7].

### 2.2. Geometric and Model-Based Methods

These methods extract facial key points as the global features such as eyes, nose, mouth, etc. The intra-ocular distance is used to find the head pose and gaze of the person. It is desirable to consider the trade-off between the number of key points and accuracy [8]. Active Shape model and Active Appearance model are used to find the landmarks and create a deformable face shape model. These techniques are used to generate 3D face models [9]. High-accuracy applications require the extraction of a more significant number of landmarks, which increases the algorithm’s complexity at changing illumination conditions, and occlusions degrade the system’s performance further [3]. These methods determine the gaze direction by obtaining mapping functions from the features of 2D eye images [10]. These methods are quite simple but are unsuitable for many real-world applications because they have difficulty finding head movements [11]. Model-based approaches use three-dimensional geometrical relationships among the eyes, infrared light sources, and camera positions. The optical axis is estimated by connecting the line between the cornea and pupil center from which the visual axis is obtained from user-dependent offset angles [12,13]. The visual axis is more sensitive to focal length, relative positions among the camera, display screen, and offset angles, and it is still a challenging task to obtain gaze direction estimation accurately by using custom-made devices with one camera and a small display screen. Most existing gaze estimation techniques include model-based or feature-based techniques [14,15,16]. The geometric models of the eyeball and its environment extract eye features (corneal infrared reflections, pupil center and iris contours) to fit the model. However, these methods are challenging to build and calibrate. Researchers classify the existing geometric characteristics-based gaze estimation techniques into shape-based (centroid points and primitive geometric shapes such as ellipse, rectangle, circle) and motion-based (tracked features of objects in videos) techniques [3].

### 2.3. Deep Learning-Based Methods

Researchers used deep learning techniques for both feature extraction and classification. One of the most popular deep learning techniques is Convolutional Neural Network (CNN). CNN is used for pose estimation, but the accuracy is not up to the mark in real time. The technique was improved by extracting the local features from the image, and then, CNN was applied for classification [9,17,18].

Table 1 shows the general description of the existing HPGE techniques. These non-intrusive head and eyelid movements observation systems fit very well to the requirements of the objectives of this research work. Novel extensions are related to a better analysis of the facial dynamics and to their correlation with different mood and emotion states.

This work focuses on the hybrid approach, which combines appearance-based and geometric-based pose and gaze estimation methods. First, the Viola–Jones algorithm extracts the global features such as eyes, and then Zernike moments are used to extract the local features from the face image and eyes for HPGE.

## 3. Methodology

**Figure 1:**
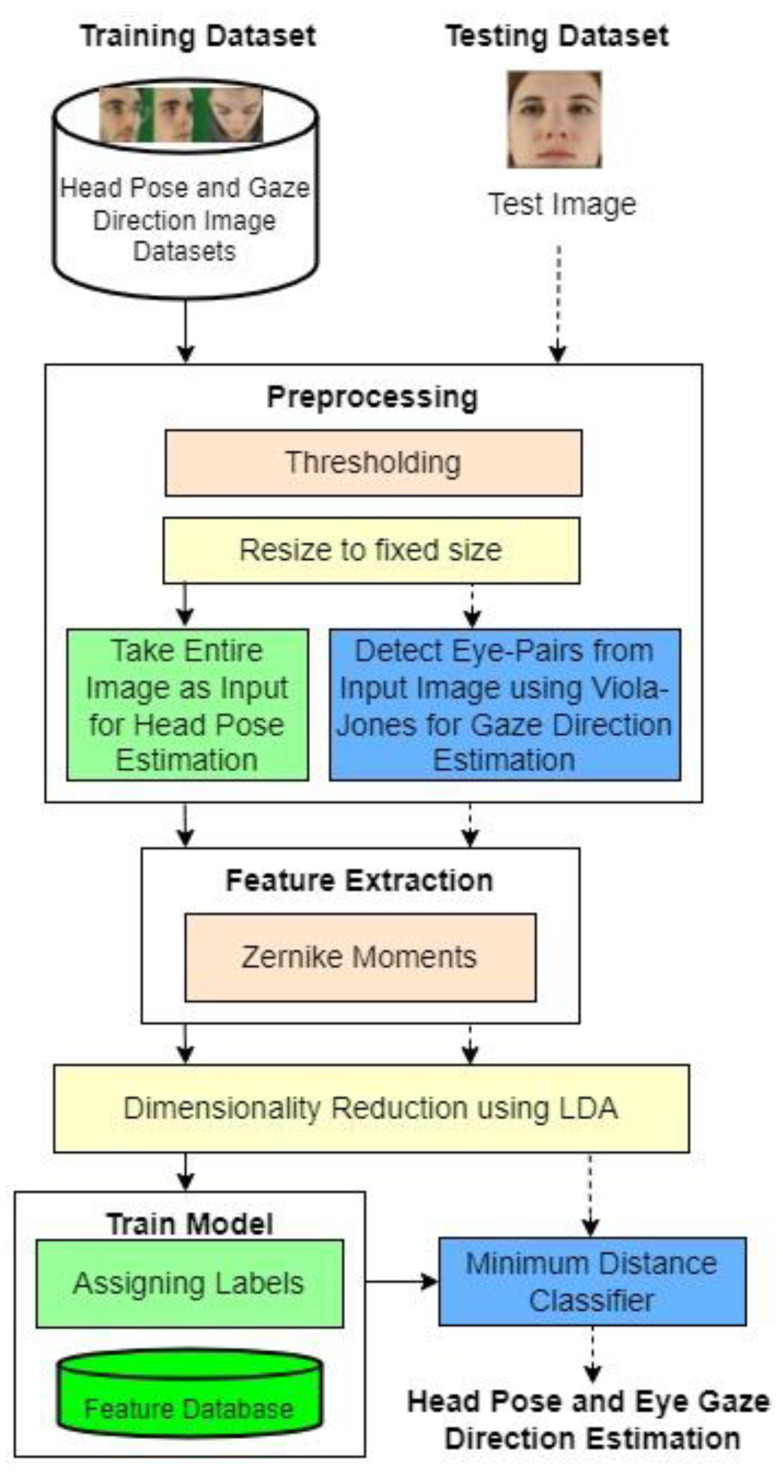
Overall head pose and gaze direction estimation system.

The overall flowchart of the proposed automatic head poses and gaze direction estimation system is shown in Figure 1. The proposed approach consists of the training phase and the testing phase. The preprocessing, feature extraction using Zernike moments, and dimensionality reduction steps are typical for the train and test phases. The minimum distance metric classifier is used to find the head pose and gaze direction estimation.

### 3.1. Preprocessing and Eye Pair Detection

The input images considered for this work are color (RGB) images. RGB color-based image has high-intensity value pixels versus the gray color image. To improve the computation speed and reduce the processing time, the color images are converted to grayscale images. For head pose estimation, there is no need to perform the face detection algorithm and pass the preprocessed image directly as the input to the feature extraction step because the Viola–Jones algorithm cannot detect faces in extreme poses. The image is resized to 256 × 256. In gaze direction estimation, the Viola–Jones algorithm detects the eye pairs of the face image [33,34]. The Viola–Jones algorithm detects the eye pairs very fast and accurately. The detected eye pairs are resized to 100 × 100.

### 3.2. Feature Extraction

For any successful recognition system, the representation of facial features is essential. The facial features are extracted using Zernike Moments (ZM) computation, and implementation is straightforward. First, the detected face features are extracted using the ZM feature extraction technique. It is defined as a unit disk space, determining the disk center by calculating the centroid of an image. The main advantage of using these moments is simple translational and scale-invariant techniques, which give high accuracy for the detailed shapes. From this, the system obtains a high dimensional feature vector.

#### 3.2.1. Zernike Moments Feature Computation

Zernike moments was initially introduced in the 1930s by Fritz Zernike. Later, it was adapted to images in the 1980s by Teague. Previously, hue moments were used as shape descriptors. These Zernike moments are based on orthogonality functions. ZM gives the shape information along with the pixel intensities, which improves the performance.

The Zernike moments are obtained from the transformed unit disk space for the extraction of shape descriptors invariant to translation, rotation, and scale, along with skew and stretch used for the feature extraction process to preserve more shape information [35,36]. These moments are the set of complex polynomials that form a complex orthogonal set over the interior of the unit circle, i.e., *p*^2^ + *q*^2^ ≤ 1. ZM are the projection of the image function on some orthogonal basis function introduced [37].

Let *I*(*p*,*q*) be the input image of size P X Q. By decomposing *I*(*p*,*q*) into the ZM basis function, the set of polynomials is denoted by {*Vnm*(*p*,*q*)}. These polynomials can be represented in Equations (3) and (4) [24].

(3) $$Vnm\left(p,q\right)=Vnm\left(rho,theta\right)=Rnm\left(rho\right)*e^{\left(j*m*theta\right)},theta\leq 1$$

(4) $$rho\left(p,q\right)=\sqrt{\left(p^{2}+q^{2}\right)},\;\;theta\left(p,q\right)=tan^{-1}\left(\frac{q}{p}\right)$$

where *n* specifies the polynomial order used to control the number of coefficients. The value of *n* should be a positive integer or zero represents the number of iterations. The value of *m* should be taken as positive or negative integers. *n* and *m* values should satisfy the conditions in Equation (5).

(5) $$\begin{array}{l} \left(a\right)\;n\;\epsilon \;Z^{+}\;\\ \left(b\right)\;n-\left|m\right|\;is\;even,and\;\\ \left(c\right)\left|m\right|\leq n \end{array}$$

The *Rnm*(*rho*) is the Zernike/Radial basis polynomial, which is defined as Equation (6)

(6) $$Rnm\left(rho\right)=\left(\overset{\frac{\left(n-|m|\right)}{2}}{\underset{s=0}{\sum }}\frac{\left(-1^{s}\right)*rho^{n-2s}*\left(n-s\right)!}{s!*\left(\frac{n+\left|m\right|}{2}-s\right)!*\left(\frac{n-\left|m\right|}{2}-s\right)!}\right)$$

where *rho* represents the length of the vector from the origin to the pixel (*p*,*q*) (image pixel radial vector). Theta represents the angle between the X-axis and the vector rho in the counter-clockwise direction.

#### 3.2.2. Zernike Moments Calculation

1. Take an entire image *I*(*p*,*q*) of size 256 × 256 as input.
2. Determine the center of the image.
3. Create a square window of minimum size (100 × 100 for gaze estimation, 64 × 64 for pose estimation) to focus the head region without distortion.
4. Place a square window in the original input image so that the image center should be the center of the square window.
5. Determine the order (*n*, *m* values) by satisfying the condition in Equation (5).
6. Calculate ZM basis function to generate the polynomials by using Equations (4) and (6).
7. Calculate complex Zernike moments from the image as Equation (7) using the basis function.

(7) $$Vnm\left(p,q\right)=Vnm\left(rho,theta\right)=Rnm\left(rho\right)*e^{\left(j*m*theta\right)},theta\leq 1$$

8. Reconstruct the shape using the basis function and complex Zernike moments.

Here, input image is resized to 256 × 256. For pose estimation, divide the input image into 16 sub-images, each of size 64 × 64. Create a disk with radius as 64 and center at (32,32). Features are extracted using the 8th order Zernike moments (orthogonal polynomial), and they produce 25 descriptors/features from each sub-image. Higher-order moments are numerically unstable, sensitive to noise and will obtain a ringing effect at the edges. Therefore, there are a total of 16 × 25 = 400 descriptors that are uncorrelated, scale, translation, and rotation-invariant features in the image. For gaze estimation, after the Viola–Jones algorithm, eye images are resized to 100 × 100. In this case, a disk of radius 100 center at (50,50) is created. Total features extracted from the 8th order ZM are 25 features from each eye image.

### 3.3. Dimensionality Reduction

After the feature extraction step, 400 features for pose estimation and 50 features for gaze estimation are extracted using 8th order Zernike moments. To improve the system performance, principal component analysis (PCA) and linear discriminant analysis (LDA) are used for dimensionality reduction. These two methods are appearance-based methods that use minimum distance classifier (Euclidean distance) for classification. In this work, Mahalanobis distance is used to improve the accuracy for multivariant data (different variations in the data) [25].

The main difference between PCA and LDA is that PCA dimensionality reduction will take place based on the selection of eigen values. We must choose the top eigen values/eigen vectors. The data compression and performance depend on the number of eigenvectors chosen. LDA orders the dimensions according to class separability. PCA takes more time to recognize the expressions, and the classification rate is also less than LDA.

### 3.4. Head Pose and Gaze Direction Estimation

After assigning labels in the training phase, the low dimensional feature vectors of the trained images are stored in the database. Then, the system estimates the head pose using the minimum distance metric (Euclidean distance) based on these features. Minimum distance class is considered as a result of reducing the false-positive rate.

## 4. Results and Discussion

The experiment was conducted on collected images with the proposed Zernike moment-based framework applied to different sizes, invariant illuminations, and occlusions [38]. The images were taken from Aberdeen, MIT-CBCL (Massachusetts Institute of Technology and to the Center for Biological and Computational Learning), Iranian, and IMM (Informatics and Mathematical Modelling) databases and real-time images (eight subject images are captured randomly with a low-cost and low-resolution camera with different environmental conditions in real time). First, the head pose is estimated by pan angle ranging from −90 to +90 degrees, and the values were discretized with 45 degrees step.

To find the efficiency of the proposed pose estimation technique, experiments were conducted on the listed database and real-time images. The details of the images and class are shown in Table 2. Real-time images are used to experiment on the gaze estimation technique.

A total of 1475 images were selected for pose estimation, out of which 740 images were considered for the training phase, and 735 images were considered for the testing phase randomly without any repetition of images. Experiments were conducted by considering all the database images together. Images were categorized into five classes (Left_90, Left _45, Front, Right_45, Right_90). The proposed system was evaluated based on performance accuracy and computation time.

### 4.1. Data Collection

**Figure 2:**
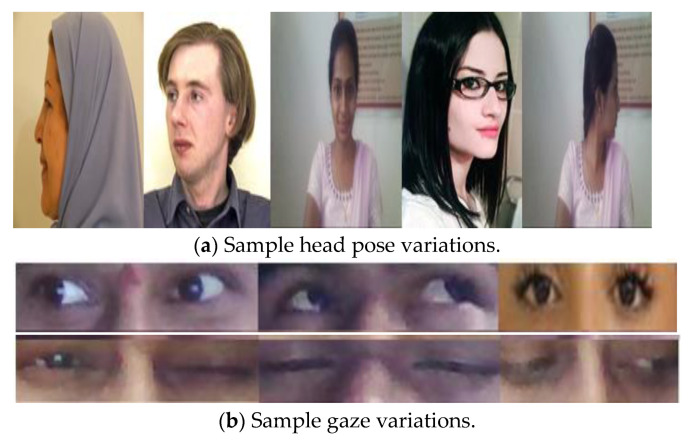
Sample head pose and gaze images.

In the Aberdeen database, each image varies in illumination, pose, facial expression and occlusion [39]. The MIT-CBCL database contains 10 subjects and 6 images per subject, one for each representing pose variation [38]. In contrast, the Iranian Face database contains 369 images of 34 human faces [40]. Each human face has 10 images representing different pose variations. Each image in the IMM face database represents poses or illumination [38,41]. The input images of different sizes and invariant illumination conditions were considered in real time, as shown in Figure 2. The details of the different pose images in the database and real time are mentioned in Table 2.

For gaze estimation, images are either taken from the frontal face databases, which differ in gaze or real-time images taken from the real-time video (divide the images into frames) captured from camera. The details of the different eye gaze directions estimated in the proposed technique are shown in Table 3.

### 4.2. Performance Evaluation for Zernike Moments Feature Extraction

**Figure 3:**
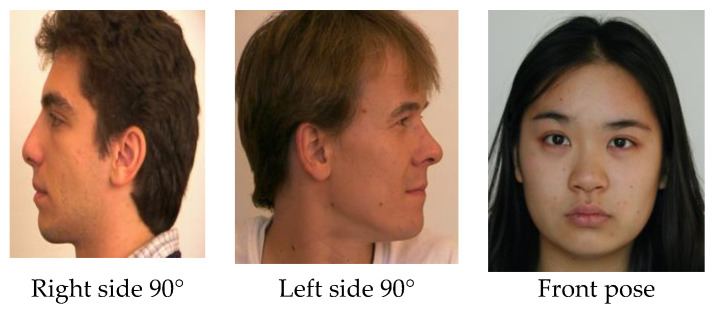
Estimation of the head pose for unknown images for different illumination conditions.

The performance evaluation was performed on the listed database and real-time images. Figure 3 shows the correctly estimated head poses for unknown real-time images.

The proposed approach gives better results in terms of accuracy and response time even at different lighting conditions, as shown in Figure 3.

**Figure 4:**
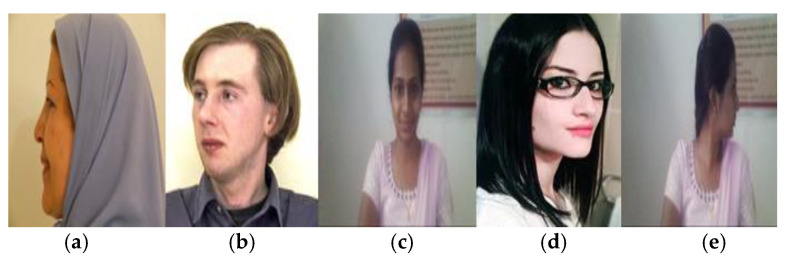
Estimation of head pose for occluded images. (**a**) Right pose; (**b**) Right pose with 45°; (**c**) Front pose; (**d**) Left pose with glasses; (**e**) Left pose.

It also works well even in occlusion conditions. Figure 4 shows that the proposed system correctly estimates the head poses with occlusions.

**Figure 5:**
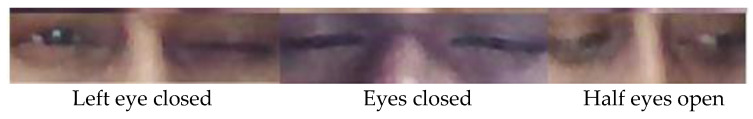
Estimation of gaze direction for varying illumination and occlusion conditions.

The gaze direction estimation is also performed with good accuracy in different illumination and occlusion conditions as shown in Figure 5.

The system’s response time is calculated from the images of the database, as shown in Table 4.

The dimensionality reduction techniques (PCA and LDA) are performed on the feature vector to obtain accurate information and increase the operational speed. LDA takes less time to estimate when compared to PCA. The time required for estimating each gaze direction using PCA and LDA is shown in Table 5.

Table 4 and Table 5 show the performance of both PCA and LDA techniques. LDA gives better classification results compared to PCA. The accuracy is performed on images of the database or unknown images using PCA and LDA, as shown in Table 6.

Real-time head poses and gaze direction estimation plays a key role in many real-time applications. In this approach, the eye pair is detected using the Viola–Jones algorithms. Here, the Viola–Jones technique is not used for face detection because it cannot detect extreme pose conditions. Thus, the entire face image is passed as an input to the feature extraction technique. The Zernike moment technique is used for feature extraction, scale, translation-invariant and illumination-invariant features. For dimensionality reduction, both LDA and PCA are used. Compared to both, LDA gives better results in less response time. The minimum distance metric method estimates the pose and gaze direction. However, it will not estimate the correct results when blurring the images. The performance is evaluated by taking the images from the public databases or real-time images.

The proposed approach attains an accuracy of 87% using LDA and 79% using PCA for estimating the head pose and 85% using LDA and 77% using PCA for gaze direction estimation, as shown in Table 6. These experimental results show that pose and gaze direction is estimated with less response time and more accuracy on real-time images even if there is a change in illumination conditions, background, and occlusion.

## 5. Conclusions

We successfully estimated the head pose and gaze direction from the proposed Zernike moment-based approach with an accuracy of 85% after using LDA. While the existing system cannot estimate the results correctly in low-resolution blurred camera conditions, our proposed method still seems promising. Furthermore, even the most accuracy-significant real-time applications, such as driver assistance or interactive system, are operable at an accuracy of 90%. In this situation, our findings closely approach those benchmarks. Furthermore, it reveals that the proposed method can be suitable for implementing the system invariant to scale, translation, illumination and blurring conditions for performance enhancement.

## Figures and Tables

**Table 1:** Description of existing techniques.

Technique	Description
Head and facial movement analysis	Example 1: An infrared active sensor is used to detect both pupil and head motions in variable light conditions. By using the Kalman filter, facial features are tracked, and a smoothening of the motion of the features is ensured. The Gabor wavelets are used for fast feature detection [19]. Example 2: First, the face and eyes are detected using the Adaboost classifier and then passed through a Gabor filter. The output is normalized and passed to a data-driven classifier, a support vector machine (SVM) [20,21]. Example 3: Human face and facial features are detected using Haar Wavelets with Adaboost cascade algorithm. Then, eye closing is measured by analyzing the optical flows in the particular region [13,22].
Eye closure, blink rates	Example 1: The PERCLOS video-based system calculates the number of eyelid closures. It measures this within 1 to 3 min intervals. A special algorithm uses this number to estimate the person’s drowsiness. A person under drowsiness generally has a longer eye closure than an alert person. This system has been validated in real on-road driving and with the Psychomotor Vigilance test (PVT) [23,24,25]. Example 2: Kinect cameras (passive stereo pair) are used to capture video of the human’s head to generate the 3D pose in real time. The persons face (±1 mm, ±1 deg) as well as the eye gaze direction (±3 deg), blink rates and eye closure are measured [23,26].
Eye blink detection approach	S multi-sensor system was developed to integrate eye lid camera (measure eye blinks) and other monitoring parameters (a steering grip sensor, a lane tracker). All signals are integrated to perform the task [27,28,29,30]
Multiple measures approach	An example of alertness-monitoring technologies comprises the MINDS system and eye-gaze system to estimate the head position and gaze, two potential fitness-for-duty systems (Safety Scope and Mayo Pupillometry system). Using this system, one can measure various parameters such as physiological, behavioral and subjective sleepiness measures. Moreover, all these parameters were integrated using a neural-fuzzy hybrid scheme [31,32].

**Table 2:** Pose information on each dataset.

Database Name	Total No of Images	Left_90	Left_45	Front_0	Right_45	Right_90
Aberdeen	687	16	15	656	-	-
MIT-CBCL	59	10	12	16	10	11
Iranian	369	106	41	78	72	72
IMM-Data	240	-	47	151	42	-
Real-Time	120	12	40	20	35	13

**Table 3:** Eye gaze directions estimated in proposed system.

Different Gaze Directions		
Lower left	Lower middle	Lower right
Middle left	Middle (Front)	Middle right
Upper left	Upper middle	Upper right
Eyes Not Open	Half eyes open	One eye open

**Table 4:** Elapsed time to estimate each head pose using PCA and LDA.

Elapsed Time (ms)	PCA	LDA
Left_90	22.5	21.6
Left_45	22	21.5
Front_0	21.9	21.5
Right_45	21.4	21.4
Right_90	21.8	21.6

**Table 5:** Elapsed time to estimate the gaze direction using PCA and LDA.

Elapsed Time (ms)	PCA	LDA
Eyes Open	7.7	7.5
Eyes Closed	7.61	7.45
Half Eyes Open	7.43	7.37
One Eye Open	7.66	7.48
Right	7.7	7.7
Left	7.8	7.4

**Table 6:** Performance analysis of proposed system using PCA and LDA.

Accuracy	PCA	LDA
Proposed Head Pose Estimation	79%	87%
Proposed Gaze Direction Estimation	77%	85%

## Data Availability

All the data relevant to this work are available in the manuscript. Raw data and material are available on request.
